# Diagnosis of COVID-19 using multiple antibody assays in two cases with negative PCR results from nasopharyngeal swabs

**DOI:** 10.1007/s15010-020-01497-2

**Published:** 2020-08-12

**Authors:** Marianna Theresia Traugott, Wolfgang Hoepler, Tamara Seitz, Sebastian Baumgartner, Mario Karolyi, Erich Pawelka, Emanuela Friese, Stephanie Neuhold, Hasan Kelani, Florian Thalhammer, Alexander Zoufaly, Hermann Laferl, Judith Helene Aberle, Christoph Wenisch, Elisabeth Puchhammer-Stöckl, Karin Stiasny, Stephan Walter Aberle, Lukas Weseslindtner

**Affiliations:** 1 4th Medical Department, Department of Infectious Diseases and Tropical Medicine, Kaiser-Franz-Josef Hospital, Vienna, Austria; 2 Division of Infectious Diseases and Tropical Medicine, Department of Medicine I, Medical University Vienna, Vienna, Austria; 3 Center for Virology, Medical University of Vienna, Kinderspitalgasse 15, 1090 Vienna, Austria

**Keywords:** SARS, Coronavirus, Antibodies, Immunoassay, Rapid test, PCR negative

## Abstract

We report of two cases of progressed COVID-19 with negative PCR tests from nasopharyngeal swabs, in whom diagnosis was made by different antibody assays, including a lateral flow rapid test and multiple commercial ELISAs, finally confirmed by comprehensive serological assays. These cases highlight that commercial ELISAs and even rapid tests might significantly aid the diagnosis of COVID-19, particularly, if a combination of serological assays is used with a specific clinical question, in severely ill patients after seroconversion and when comprehensive serological methods are used for confirmation.

**Electronic supplementary material:**

The online version of this article (10.1007/s15010-020-01497-2) contains supplementary material, which is available to authorized users.

## Introduction

SARS-coronavirus 2 (SARS-CoV-2) is a novel coronavirus causing coronavirus disease 2019 (COVID-19) [1]. After first cases were described in Wuhan, China in December 2019, the virus has been rapidly spreading all over the world, causing a global pandemic with massive consequences to the health-care systems worldwide [2].

Immediately after discovery of the virus, polymerase chain reaction (PCR) from the upper airway (nasopharyngeal swabs) became the main diagnostic test method [3]. However, false-negative PCR results have been described by several studies [4–6]. Additionally, chest computer tomography (CT) scans have diagnostic value in advanced disease, which even caused a change of the original case definition in China, using clinical symptoms and radiological signs rather than PCR as main diagnostic criteria [7, 8]. Typical radiological signs for COVID-19 are peripheral, subpleural ground glass opacities, often located in the lower lobes [7].

As additional diagnostic tool, serological assays for SARS-CoV-2-specific antibodies have recently become available. While there is a high public demand to use these tests in the common population to identify individuals with probable immunity, we recently showed that a combination of multiple serological assays might have excellent performances in diagnosing SARS-CoV-2 infections in symptomatic, hospitalized, and severely ill patients [9].

Here, we report of two cases, in which COVID-19 was diagnosed only by serological assays (including a lateral flow rapid test) in patients who displayed progressed disease and PCR negative swabs from the upper respiratory tract.

## Case 1

A 71 year old, male patient was transferred to our COVID-19 intensive care unit (ICU) because of rapid respiratory deterioration with high suspicion of COVID-19 based on a chest CT-scan revealing multiple, bilateral, partially confluent ground glass opacities, thickening of the interlobar septum, and the bronchial wall and small focal consolidations. The result of his SARS-CoV-2 nasopharyngeal swab was still pending at this time. The patient had a medical history of prostatic hyperplasia and arterial hypertension (treated with bisoprolol 5 mg q.d) and reported a high fever and strong retrobulbar headache for about 10 days but no cough. He had been on a cruise ship journey in the Caribbean at the end of February (he could not recall the exact dates), from where he returned via the Dominican Republic. A malaria rapid dipstick test and smear and a dengue rapid test gave negative results.

While the patient could be stabilized in our ICU, PCR testing for SARS-CoV-2 from a nasopharyngeal swab gave a negative test result (Roche^®^ COBAS 6800, CE-IVD Assay; Roche Diagnostics, Rotkreuz, Switzerland). Isolation measures, however, were maintained due to unchanged high suspicion of COVID-19. The initial laboratory results showed a normal white blood cell count (WBC) of 7500 G/l with lymphocytopenia (9%), thrombocytopenia (149 G/l), and an elevated C-reactive protein (CRP) of 140 mg/l. Antibiotic therapy with intravenous cefuroxime and azithromycin was started. A PCR out of sputum (pneumonia panel plus, Biomerieux^®^) targeting 33 different kinds of bacteria and viruses (incl. influenza A/B, Legionella, Mycoplasma) was all negative, as was the HIV test. At the 11th day post-onset of symptoms, a blood sample was sent to the Center for Virology for PCR and serology testing for tropical infectious diseases (chikungunya IgM, IgG and PCR, dengue hemagglutination inhibition assay, and Zika virus IgM and neutralization assay: all negative), as well as for SARS-CoV-2-specific serology. However, SARS-CoV-2-specific antibodies tested negative for IgM and total antibodies (both Wantai, Beijing, China) as well as for IgA and IgG (both Euroimmun, Lübeck, Germany) using enzyme-linked immunosorbent assays (ELISAs). A second nasopharyngeal swab for SARS-CoV-2-PCR was performed, but again gave a negative result (this time using an in-house SARS-CoV-2 real-time TaqMan PCR with WHO recommended primers, a probe located in the E-gene and an internal spiked-in control to exclude PCR inhibition, as described previously) [3]. No antibiotics treatment had been initiated before initial PCR testing and no gurgling with antiseptic substances was performed.

Since the patient remained stable on high-flow nasal cannula (HFNC) and inflammation markers decreased, he was transferred to a non-COVID-ICU at the 12th day after onset of symptoms (during the stay on the ICU he was never intubated). Still struggling for a diagnosis, PCR tests for SARS-CoV-2 were repeated on the 19th and the 20th day post-onset of symptoms. Both PCRs out of deep sputum (induced by NaCl inhalation) tested negative. A serum sample drawn at the 20th day post-symptom onset had arrived too late for ELISA testing at the same day. Therefore, a Wantai rapid test (Wantai, Beijing, China) was performed, which showed a positive result. On the next day, the positive test result was confirmed by the detection of SARS-CoV-2-specific IgM (ratio: 35.83), total antibodies (ratio: 8.55), IgA (ratio: > 12.00), and IgG (ratio: 8.52). It was the 10th day after hospital admission and the 20th day after onset of symptoms. Since all PCR tests continuously tested negative, antibody cross-reaction with other coronaviruses was not excluded, although seroconversion of anti SARS-CoV-2 was clearly identified by four ELISAs. To rule out infection with other coronaviruses, the last sputum sample was retested for coronaviruses OC43, 229E, HKU-1, and NL63 by PCR (using specific primers) giving negative results. Finally, an immunoblot microarray (Viramed, Munich, Germany), which concordantly detected high levels of IgM, IgA, and IgG against all viral proteins S1, S2, and nucleocapsid confirmed the presence of SARS-CoV-2-specific antibodies, and thereby SARS-CoV-2 infection.

## Case 2

A 61 year old male patient was transferred from the normal COVID-19 ward to our ICU because of rapidly increasing oxygen demand. The patient reported onset of symptoms with fever, cough, and dyspnoea 9 days earlier. The patient had been tested positive for COVID-19 by nasopharyngeal swab 6 days after onset of symptoms, performed at home by the Austrian mobile outreach COVID-19-testing-service. The same day, he was admitted to our hospital, not bringing a copy of the positive PCR result. The patient had a medical history of Hashimoto thyroiditis (treated with Euthyrox 150 µg q.d.), arterial hypertension (treated with candesartan 4 mg q.d.), and status post disc prolapse surgery. One day later (7th day post-onset of symptoms), another nasopharyngeal swab was taken, yielding a negative result (in-house SARS-CoV-2 real -time TaqMan PCR with the primers described above and using an internal spiked-in control to exclude PCR inhibition), and also SARS-CoV-2-specific IgA and IgG antibodies tested negative (both Euroimmun, Lübeck, Germany). Also in this case, there was no gurgling with antiseptic substances and no previous treatment with antibiotics.

On the day of ICU admission (day 9 after symptom onset, 3 days after hospital admission) laboratory results showed leukopenia with a WBC of 2.99 G/l with lymphocytopenia (0.64 G/l), aneosinophilia, thrombocytopenia (117 G/l), and a slightly elevated CRP of 30 mg/l. Lopinavir/ritonavir had been started already on the admission day and was continued. Oxygen was administered by HFNC with increasing FiO_2_.

On the 10th day after symptom onset, another PCR was performed with a swab from the upper respiratory tract and again gave a negative result. The respiratory situation, however, deteriorated, CRP increased to 119 mg/l, and IL-6 was 256 pg/ml. In this typical constellation, cytokine storm was suspected and Tocilizumab combined with cortisone was administered. Until that day, the treating physicians had never seen any positive SARS-CoV-2 test of the patient, but for further patient management (including antiviral therapy, cortisone therapy as well as convalescent plasma administration), a definitive written positive COVID-19 result was crucial. The patient further deteriorated on the 12th day post-onset of symptoms and had to be intubated in the early morning. The same morning, deep tracheal secretion (tracheal aspirate), a nasopharyngeal swab, and a serum sample were taken and sent to the Center for Virology. Due to the urgent need of a diagnosis, a Wantai Rapid test (Wantai, Beijing, China) was immediately performed and gave a weakly positive result. With this preliminary result indicating SARS-CoV-2 infection (especially with association of negative serology on the 7th day post-onset of symptoms), convalescent plasma could be transfused and therapy with remdesivir was started. On the following day, the same serum sample (drawn before the plasma transfusion and already tested with the rapid test) was tested using ELISA, and the positive test result by the rapid test was confirmed (ratio IgA: 3.97, ratio IgG: 1.75). Finally, an in-house neutralization assay confirmed the specificity of those antibodies. The protocol of this assay is described in Supplemental Material and Methods. PCR from the deep tracheal secretion (obtained at the 12th day post-onset of symptoms) finally tested positive (1.1 × 10^5^ copies/ml) for SARS-CoV-2, while in the nasopharyngeal swab from the upper respiratory tract, viral RNA was still undetectable.

## Conclusion

With respect to COVID-19 diagnosis, these two cases highlight important aspects: First, they demonstrate limitations of PCR-based tests from nasopharyngeal swabs even in advanced disease stages. Indeed, patients with progressed pneumonia may display lower virus concentrations in the upper respiratory tract (possibly causing false-negative PCR results from pharyngeal swabs) [10]. It has already been shown for SARS-coronavirus that virus concentrations in nasopharyngeal swabs and saliva may be significantly lower than in sputum [11, 12]. Also in SARS-CoV-2 infections, viral RNA may be still detected by PCR in sputum when nasopharyngeal swabs already test negative [13]. Therefore, (deep) sputum should be preferably used for PCR analysis when swabs test negative in patients with clinical suspicion of COVID-19.

In severely ill patients with SARS-CoV-2 infection, however, specific antibodies may also be detectable and significantly aid the diagnosis, as demonstrated in our cases [9]. Indeed, we detected significant antibody titres at the 20th and 12th day post -onset of symptoms with five different assays in both of our cases.

Second, both cases highlight the caveat that serology is not able to identify infections early after disease onset and that the time span until significant antibody levels are produced may individually vary. Of note, antibody tests gave negative results in the patients at the 11th and the 7th day post-onset of disease, respectively, but these early acquired samples, nonetheless, facilitated us to subsequently observe seroconversion, thus providing evidence for the course of the infection. Importantly, serological results should always be interpreted in conjunction with the date of symptom onset. However, the detection rates of different immunoglobulins, in relation to the interval since symptom onset, may vary among different assays depending on the configuration of the respective tests [13–15].

Third, these two cases demonstrate that even rapid tests have potential to aid COVID-19 diagnosis. Importantly, there are large numbers of lateral flow rapid tests currently on the market, and each of these tests has to be thoroughly evaluated for the specific setting of its possible use [16]. While certain rapid tests may not have sufficient sensitivity and specificity for a widespread use in the population and for large seroprevalence studies (especially when used as single tests), other tests may be particularly useful in distinctive patient cohorts under distinct circumstances. For the rapid test which we applied in our two cases, we recently demonstrated a sensitivity of 80% between the 6th and 10th day and of 100% after the 11th day post-onset of symptoms in hospitalized patients with SARS-CoV-2 infection, while the specificity of the test was 98% [9]. Since patients with severe symptoms may display higher antibody levels than patients with mild or asymptomatic SARS-CoV-2 infections, the same rapid test may, thus, not have the same diagnostic power in patients with mild disease (and lower antibody levels). Thus, also the ELISA could display significantly lower performances in seroprevalence studies covering a large part of individuals with mild symptoms not requiring hospitalization [15].

The presented cases also highlight that comprehensive serological methods (immunoblot and/or neutralization assays) are required to finally confirm positive test results by SARS-CoV-2 ELISAs/chemiluminescent immunoassays (CLIAs) or lateral flow assays (rapid tests), particularly when the diagnosis of COVID-19 is solely based on serology. Although the early evaluation studies demonstrate acceptable specificities for commercial ELISA/CLIA test systems, the possibility of unspecific or cross-reactions with other coronaviruses has to excluded in clinically relevant cases by antibody tests with highest specificity [17–19].

In summary, these cases demonstrate the potential of serological assays for diagnosing acute SARS-CoV-2 infections, especially when there is a high grade of clinical suspicion for COVID-19 and antibody testing is repeatedly performed during progressing infection. Furthermore, our cases indicate that antibody tests may additionally be a useful tool for fast decision-making in patient management. Reactive antibody tests indicate that the sensitivity of PCR-based tests from nasopharyngeal swabs may be limited in progressed COVID-19 cases and that antibody assays may provide negative results early after the infection, important aspects that should be considered in the diagnosis of COVID-19.

## Electronic supplementary material

Below is the link to the electronic supplementary material.

Supplementary file1 (DOCX 13 kb)

## Data Availability

Data will be made transparent upon publication.

## Abbreviations

Ab: Total antibody
COVID-19: Coronavirus disease 2019
CLIA: Chemiluminescent immunoassay
CRP: C-reactive protein
CT: Computer tomography
ELISA: Enzyme-linked immunosorbent assay
HFNC: High-flow nasal cannula
ICU: intensive care unit
Ig: Immunoglobulin
SARS-CoV-2: Severe acute respiratory syndrome coronavirus 2
PCR: Polymerase chain reaction
WBC: White blood cell count
