# Supplementary material for: Diagnosis of COVID-19 using multiple antibody assays in two cases with negative PCR results from nasopharyngeal swabs
Source: Infection. 2020 Aug 12;49(1):171–5. doi: 10.1007/s15010-020-01497-2 (PMC7851003; doi:10.1007/s15010-020-01497-2)
Supplement: Supplementary file 1 — Supplementary file1 (DOCX 13 kb) [file 15010_2020_1497_MOESM1_ESM.docx]

**Supplemental Material and Methods**

The SARS-CoV-2 neutralization test (NT) was based in principle on our NT for measles virus [1]. Of note, instead of Vero-SLAM cells we used Vero-E6 cells. Furthermore, we observed that incubation time until cytopathic effects (CPE) was shorter for SARS-CoV-2 than for MeV. Briefly, two-fold serial dilutions of heat-inactivated serum samples were incubated with 50-100 TCID50 of infectious SARS-CoV-2 (GISAID/EPI_ISL_438123/hCoV-19/Austria/CeMM0360/2020) for 1h at 37 °C under Biosafety Level 3 conditions. The mixture was added to Vero E6 (ATCC ® CRL-1586) cell monolayers and incubation was continued for two to three days at 37°C. Virus neutralization was assessed by assessment of CPE. NT titres were expressed as the reciprocal of the serum dilution that protected against virus-induced CPE. NT titres ≥10 were considered positive.

**Reference:**

1. Semmler G, Griebler H, Aberle SW, Stiasny K, Richter L, Holzmann H et al. Elevated CXCL10 serum levels in Measles virus primary infection and reinfection correlate with the serological stage and hospitalization status. J Infect Dis. 2020. doi:10.1093/infdis/jiaa326.
